# Effects of Virgin Coconut Oil-Enriched Diet on Immune and Antioxidant Enzymatic Activity, Fat and Vitellogenin Contents in Newly Emerged and Forager Bees (*Apis mellifera* L.) Reared in Cages

**DOI:** 10.3390/insects14110856

**Published:** 2023-11-03

**Authors:** Simona Sagona, Francesca Coppola, Elena Tafi, Caterina Orlando, Chiara D’Onofrio, Chiara Benedetta Boni, Lucia Casini, Lionella Palego, Laura Betti, Gino Giannaccini, Antonio Felicioli

**Affiliations:** 1 Department of Pharmacy, Pisa University, Via Bonanno 6, 56126 Pisa, Italy; caterina.orlando@unipi.it (C.O.); laura.betti@unipi.it (L.B.); gino.giannaccini@unipi.it (G.G.); 2 Department of Veterinary Sciences, Pisa University, Viale delle Piagge 2, 56124 Pisa, Italy; francesca.coppola@vet.unipi.it (F.C.); chiarabenedetta.boni3@gmail.com (C.B.B.); lucia.casini@unipi.it (L.C.); antonio.felicioli@unipi.it (A.F.); 3 CREA Research Centre for Agriculture and Environment, Via di Corticella 133, 40128 Bologna, Italy; elena.tafi@crea.gov.it; 4 Biosensor Technologies, Austrian Institute of Technology GmbH, Konrad-Lorenz Straße, 24, 3430 Tulln, Austria; ch.donofrio.agr@gmail.com; 5 Department of Clinical and Experimental Medicine, Pisa University, Via Savi 10, 56126 Pisa, Italy; lionella.palego@unipi.it

**Keywords:** coconut oil, honey bees, glucose oxidase, phenoloxidase, glutathione S transferase, vitellogenin, fat body, artificial diet

## Abstract

**Simple Summary:**

Bees are often fed by beekeepers with supplemented artificial diets. The formulation of an integrative diet for honey bee colonies able to prevent nutritional deficiencies is yet to be found. In this work, the effects of fat-enriched diet (coconut oil) administration in newly emerged and forager bees were tested. Fat supplementation in the honey bee diet determines an increase in newly emerged bees’ survival in the short term and an increase in fat content. Further investigations to evaluate the use of such a supplement food to prevent the fat loss of weak families during winter are desirable.

**Abstract:**

Searching for artificial diets positively affecting the survival, immune and antioxidant systems of honey bees is one of main challenges occurring in beekeeping. Among nutrients, lipids play a significant role in insect nutrition as structural components in cell membranes, energy sources and reserves, and are involved in many physiological processes. In this context, the aim of this work was to investigate the effect of 0.5% and 1% coconut oil-enriched diet administration on newly emerged and forager bees survival rate, feed intake, immune system, antioxidant system and both fat and vitellogenin content. In newly emerged bees, supplementation with 1% coconut oil determined a decrease in feed consumption, an increase in survival rate from the 3rd to 14th day of feeding, a short-term decrease in phenoloxidase activity, an increase in body fat and no differences in vitellogenin content. Conversely, supplementation with 0.5% coconut oil determined an increase in survival rate from the 3rd to 15th day of feeding and an increase in fat content in the long term (i.e., 20 days). Regarding the forager bee diet, enrichment with 0.5% and 1% coconut oil only determined an increase in fat content. Therefore, supplementation with coconut oil in honey bee diets at low percentages (0.5 and 1%) determines fat gain. Further investigations to evaluate the use of such supplement foods to prevent the fat loss of weak families during winter are desirable.

## 1. Introduction

The Western honey bee (*Apis mellifera*) is a pollinator playing a very important role in the world economy for both pollination and bee products [1]. In recent decades, bees have been coping with several adversities such as pathogens, the use of pesticides, climate change and changes in land use [1]. In this context, improving bee immunity status through the administration of enriched artificial diets could be a useful tool to strengthen colonies and combat challenges to bee health [2]. The innate bee immune system can be divided into social and individual immune systems [3]. The social immune system mainly consists of hygienic behaviours and the activity of glucose oxidase, which is involved in the conversion of glucose into gluconic acid and hydrogen peroxide, with antibacterial activity [4,5]. The individual immune system includes the action of haemocytes, antimicrobial peptides and the phenoloxidase enzyme, which is involved in the melanin production [6]. Melanin is the final product of an enzymatic cascade starting from the activation of pro-phenoloxidase by a serine protease and is involved in pathogen encapsulation and nodule formation [6,7]. Among the molecules with immunity functions, vitellogenin binds and/or transports the pathogen-associated pattern molecules (i.e., lipopolysaccharide, peptidoglycan and yeast zymosan), and can provide haemocytes with the necessary zinc for their immune function [8,9]. Vitellogenin is synthesized by fat bodies and regulates honey bee aging by its oxidation potential [9,10]. The antioxidant enzymes glutathione S-transferase, superoxide dismutase and catalase also play a role in bee wellness, being able to scavenge radical chemical species [11,12,13]. In recent decades, several studies on the effects of the administration of artificial diets on bee immune and antioxidant systems, survival and feed intake have been performed [14,15,16]. The administration of 1,3-1,6 β-glucans (carbohydrates) can affect survival rate, phenoloxidase activity and DWV viral load with a dose-dependent effect [16,17]. Moreover, protein diets resulted in effects on honeybee physiology, survival rate, phenoloxidase and glucose oxidase activity with an age-dependent trend [15,18,19,20,21,22,23,24]. Sagona et al. [25] also observed that a natural amount of dietary hydrolysable tannins can weakly affect bee wellness, whereas high levels can have a negative impact on honey bee immunity and metabolism leading to a reduction in lifespan. A higher content of protein and glutathione, and higher peroxidase, catalase and glutathione transferase activity were observed in newly emerged worker bees in colonies supplemented with vitamin C compared to control colonies [26]. According to Stabler et al. [27], a balanced protein/lipid ratio of 1.25:1 in the nurse bee diet is necessary. Lipids play a significant role in insect nutrition as structural components in cell membranes, as an energy source and in reserves, and are involved in many physiological processes (i.e., development, reproduction and the biosynthesis of hormones and pheromones) [10]. Pollen is the main lipid source for bees and the dietary fat concentration is usually ~1% [28]. A high-fat diet induces obesity and alterations in the bee gut microbiota profile, resulting in metabolic disorders [29]. Diet enrichment with arachidonic acid (i.e., a fatty acid) has been reported to improve the activity of immune enzymes (i.e., phenoloxidase, antitrypsin and lysozyme) in honey bees [30]. Manning et al. [31] observed in 27 different plant- and fish-based oil-enriched diets tested that only coconut oil- and linseed oil-enhanced pollen diets were significantly more consumed by honey bees than a pollen diet. Coconut oil contains high amounts of fats present in pollen such as decanoic acid, dodecanoic acid, myristic acid and palmitic acid, while a low amount of linoleic acid is present [28,31]. Coconut oil is composed of medium-chain fatty acids (MCFAs), which correspond to 64% of the total fat (of which 40–50% is made up of lauric acid), and contains phospholipids, tocopherol and other minor constituents [32]. However, no data are available on the effects of this oil on bee welfare and immune system. Therefore, the aim of this work was to investigate the effects of virgin coconut oil-enriched diets on newly emerged and forager bee survival rates, their feed intake, immune system (i.e., glucose oxidase and phenoloxidase activities), antioxidant system (i.e., glutathione S-transferase activity) and fat and vitellogenin contents.

Newly emerged bees were selected since the highest consumption of pollen occurs from emergence to 8–10 days old, while foragers bees, as their ontogeny, depend on food availability and they consume only small amounts of pollen [33,34]. Therefore, since pollen represents the main source of fatty acids for adults, we predict that fat diets could impact the wellness parameters more in newly emerged bees than in foragers.

## 2. Materials and Methods

### 2.1. Honey Bee Sampling and Rearing

Honey bees were sampled from four hives of the experimental apiary of the Department of Veterinary Sciences of Pisa University (43°40′51.45″ N 10°20′50.96″ E). We managed to obtain bees with the same family strength (adult/brood), queen’s age (2 years old), as well as the same treatment against Varroa mite and the absence of symptoms of principal honeybee diseases, such as American foulbrood, deformed wings and diarrhoea. In July 2019, a pool of 250 newly emerged and 250 forager honeybees were randomly collected from four hives. From the same hives, 25 nurse and 25 guardian bees (external control) were also randomly collected to compare their enzymatic activity, fat and vitellogenin content with that of experimental bees sampled as newly emerged and supposed to become a nurse and guardian after 5 days (T5) and 20 days (T20), respectively, of experimental caging. Newly emerged bees were sampled from sealed brood frames without adult bees. All newly emerged bees were sampled just after their emergence inside a swarm box. Workers fed larvae were sampled as nurse bees. The guardians were sampled as worker bees that showed aggressive behaviour towards the beekeeper after closing and reopening the entrance door of the hive. Worker bees with pollen loads flying in front of the hive were sampled as forager bees. Old foragers with fringed wings were discarded.

All nurse and guardian bees collected were immediately stored at −20 °C after collection until analysis. For newly emerged and forager bees, 25 bees for each imago age were immediately stored at −20 °C after collection until analysis and were used as T0. Forager bees at T0 were also used as an external control. The remaining bees for each imago age were randomly distributed in 9 Plexiglas cages (11 cm × 13 cm × 6.5 cm), 3 cages (replicates) per diet each containing 25 bees and reared at a controlled temperature (30 ± 2 °C) and natural dark/light cycle [25]. Cages were equipped with a piece of wax comb and two tip-less syringes (5 mL), one for providing feed and one for water.

Cages were grouped according to the three administered diets: candy, produced with 95% powdered sugar and 5% water (internal control diet); candy enriched with 0.5% (*w*/*w*) commercial coconut oil; and candy enriched with 1% (*w*/*w*) commercial coconut oil. Diets and water were administrated ad libitum to honey bees and daily renewed.

A bio-virgin coconut oil (i.e., obtained from the fresh, mature kernel of the coconut by mechanical or natural means, with or without the use of heat and without undergoing chemical refinement [35]) containing 92 g/100 mL of fat (86 g saturated, 5 g monounsaturated and 1 g polyunsaturated) was used. According to the label, the product did not contain carbohydrates, proteins or salt.

### 2.2. Feed Intake and Survival Rate

Feed intake was measured daily by weighing each syringe in each diet group. The mean daily individual consumption was calculated as the ratio of the quantity of diet consumed in each cage to the number of live bees. The coconut oil intake was calculated by feed intake and the percentage of coconut oil present in the diet. The surviving bees were counted daily throughout the investigation period and the dead bees were removed from cages. On the 5th day (T5) of feeding, 6 newly emerged and 6 forager bees for each diet group (2 bees per cage) were collected and stored at −20 °C until analysis. On the 20th day (T20), all the surviving newly emerged bees were collected and stored at −20 °C until analysis. Newly emerged and forager bees collected for each diet group at T5 and T20 were used for both enzymatic activity, fat and vitellogenin content analysis. Due to the high mortality rate recorded in forager bees, data collection and rearing were stopped on the 5th day.

### 2.3. Enzymatic Activity

All chemicals were purchased from Sigma Aldrich (St. Louis, MO, USA).

For glucose oxidase analyses, a protein extract of bee heads was used. A single head in n = 6 replicates per diet group was prepared as follows. Each sample was weighed before protein extraction; then, 150 µL of 100 mM phosphate buffer pH 7.2 with 1% (*v*/*v*) Triton X-100 was added. Samples were homogenized using a Teflon pestle. The ensuing supernatants were collected, and 150 µL of 100 mM phosphate buffer pH 7.2 was added to pellets. The supernatants derived from this second extraction were mixed with those previously collected, and the protein concentration of each extract was determined using Bradford’s reagent [36], using γ-globulin to create the standard curve.

Next, glucose oxidase was measured according to Sagona et al. [25]. Absorbance data were obtained at λ = 352 nm, at times 0 and 120 min, after the addition of diaminobenzidine (DAB) (0.18 mg/mL) and HRP (horseradish peroxidase) (0.02 mg/mL). Values were expressed as U/µg of proteins.

For phenoloxidase investigation, the protein extract of the bee thorax was used. A single bee thorax in n = 6 replicates per diet group was prepared as follows. The protein concentration of each thorax was determined using Bradford’s reagent following the same procedure for protein extraction as was previously described for glucose oxidase analysis. Phenoloxidase was then measured according to Mazzei et al. [16]. Absorbance data were obtained at λ = 490 nm, at times 0 and 10 min. Values were expressed as mU/mg of protein.

For glutathione S transferase analysis, the same samples previously used to assess the phenoloxidase activity were analysed.

Glutathione S-transferase activity was investigated according to Sagona et al. [24]. Absorbance data were obtained at λ = 340 nm for 5 min. Values were expressed as mM/min/mg of tissue.

### 2.4. Fat Content

Fat content was measured from the bee’s abdomen. A single abdomen in n = 6 replicates per diet group was prepared as follows. Each abdomen was weighed and placed in a 1.5 mL test tube. A solution containing chloroform/methanol (2:1) was added to each abdomen, according to Folch et al. [37]. The lipid measure was performed using a gravimetric method with modifications [38,39]. The abdomen was homogenized with a Teflon pestle and centrifuged at 3200 rpm for 5 min. The supernatant was recovered and transferred to a new 1.5 mL test tube with a known weight. Water was added in a volume equal to half of the methanol present in the extract and the mixture was allowed to decant for 30 min. When two phases formed in the solution, the upper phase was removed and placed at −20 °C for vitellogenin determination, while the lower phase, containing lipids, was dried and weighed. Lipids are expressed as mg total lipids per mg tissue.

### 2.5. Vitellogenin Content

The protein content of the upper phase removed during the fat extraction and stored at −20 °C was quantified using the Bradford method [36] and used for the vitellogenin content analysis. Vitellogenin content was measured on 40 µL of sample diluted 1:2 using a General Vitellogenin ELISA Kit (catalogue number 0772-E0010Ge, Bioassay technology laboratory, Shanghai, China). This kit is an Enzyme-Linked Immunosorbent Assay (ELISA). The plate is pre-coated with General VG (vitellogenin) antibody. When samples containing VG are added to the plate, VG binds to the antibodies coating the wells. Then, a biotinylated General VG Antibody is added and binds to VG in the samples. Then, Streptavidin-HRP is added and binds to the biotinylated VG antibody. After incubation, unbound Streptavidin-HRP is washed away. A substrate solution is then added, and colour develops in proportion to the amount of General VG. The reaction is terminated by the addition of an acidic stop solution and absorbance is measured at 450 nm.

### 2.6. Statistical Analysis

For inferential statistics, the estimated survival rate was analysed via the Wilcoxon rank test, using the product-limit (Kaplan–Meier) method. The chi-square test with Yates correction was applied for statistical differences in daily survival. Statistics for feed intake, phenoloxidase, glucose oxidase and glutathione S-transferase activities were performed as follows: Data residues, obtained using preliminary ANOVA for more factors, were tested for normal distribution with the Shapiro–Wilk test. Data were tested also for homogeneity of variances using the Bartlett test. Since feed intake, phenoloxidase, glutathione S transferase activities, fat content and vitellogenin were not normally distributed, diet differences were assessed using the non-parametric Kruskal–Wallis H-test, followed by post hoc Mann–Whitney U-test pairwise comparisons. Since glucose oxidase activity was normally distributed it was analysed using an ANOVA test followed by Tukey’s test. Correlations between coconut oil intake and survival rate, glucose oxidase activity, phenoloxidase activity, glutathione S transferase activity, fat content and vitellogenin content were also assessed, using Spearman’s test.

All statistical calculations were performed using JMP 7 software (Cary, NC, USA: SAS Institute, 2008) [40]. The 2-tailed α-error was pre-set to 0.05.

## 3. Results

### 3.1. Feed Intake and Survival Rate

**Figure 1:**
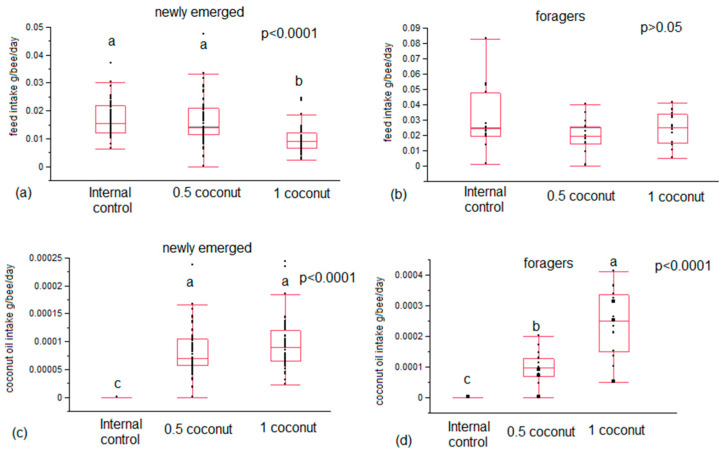
Feed intake and coconut oil intake in newly emerged (**a**,**c**) and forager (**b**,**d**) bees fed candy (internal control diet), and candy enriched with 0.5–1% coconut oil. Different letters in the graph indicate statistical differences in feed intake or coconut oil intake between diets.

In newly emerged bees, feed intake was significantly higher in bees fed both the internal control diet and the 0.5% coconut oil-enriched diet (*p* < 0.0001; χ^2^ = 50.5627; df = 2) compared to those fed 1% coconut oil (Figure 1a). In forager bees, there were no differences (*p* = 0.3057; χ^2^ = 2.3706; df = 2) in feed intake among the three diet groups (Figure 1b). Dietary coconut oil intake did not show a significant difference between newly emerged bees fed with the 0.5% and 1% coconut oil-enriched diets (*p* = 0.078; χ^2^ = 3.0921; df = 1) (Figure 1c). Conversely, forager bees fed a 1% coconut oil-enriched diet consumed significantly more coconut oil than those fed a 0.5% coconut oil-enriched diet (*p* = 0.0002; χ^2^ = 13.4748; df = 1) (Figure 1d).

**Figure 2:**
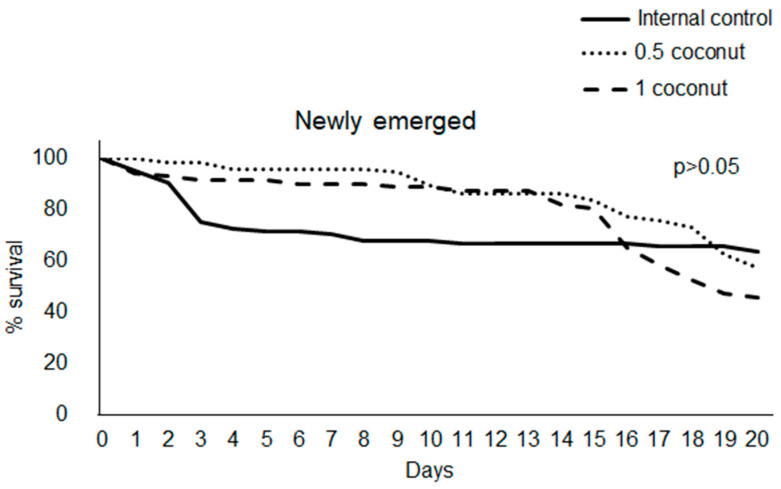
Survival rate in newly emerged bees fed candy (internal control diet) and candy enriched with 0.5 and 1% coconut oil.

**Figure 3:**
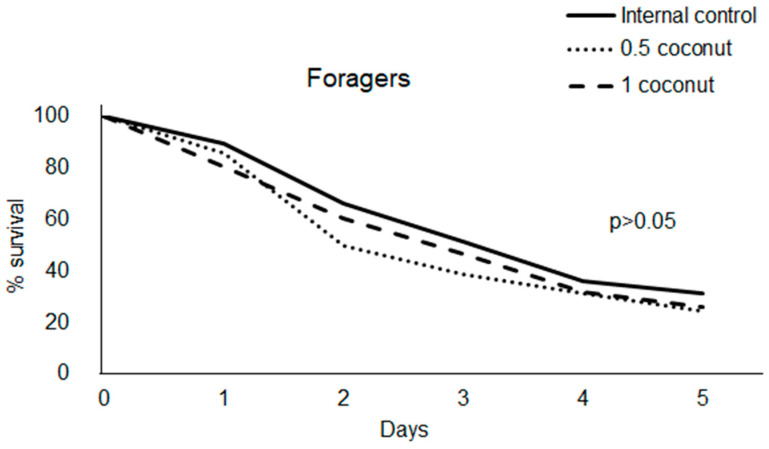
Survival rate in forager bees fed candy (internal control diet) and candy enriched with 0.5 and 1% coconut oil.

In both newly emerged and forager bees, no differences were observed in survival rate between the two experimental diet groups and the internal control one (Figure 2 and Figure 3).

In newly emerged bees, the survival rate significantly decreased in bees fed the internal control diet compared to those fed 0.5% and 1% coconut oil-enriched diets from the third day of feeding (76.5% SD 17.7, 98.5% SD 2.6, 91.5 SD 8.0, for control, 0.5% coconut and 1% coconut diets, respectively; χ^2^= 23.06, *p* = 9.83 × 10^−6^, df = 2). Statistical differences in newly emerged bee survival rates among the three groups were recorded until day 14. On day 15, the survival rate of the internal control was significantly lower than in bees fed a 0.5% coconut oil-enriched diet, while bees fed a 1% coconut oil-enriched diet showed no significant differences to the other two diet groups (68.3% SD 18.8, 82.9% SD 6.9, 80.9 SD 4.5, for control, and 0.5% coconut and 1% coconut diets, respectively; χ^2^= 6.23, *p* = 0.044, df = 2). From the 16th to the 18th day and on day 20, no statistical differences in survival rate among the three experimental diet groups were recorded, while on the 19th day, they were significantly higher in the control group than in bees fed 1% coconut oil while no differences were recorded between the 0.5% coconut oil-enriched diet and the other ones (67% SD 18.2, 58.8 SD 31.4, 48.5 SD 18.0, for control, and 0.5% coconut and 1% coconut diets, respectively; χ^2^ = 6.32, *p* = 0.042, df = 2).

The foragers showed no statistical differences in daily survival rate among the three experimental diet groups.

### 3.2. Enzymatic Activity

In newly emerged bees, neither glucose oxidase nor glutathione S transferase activities showed differences between diet groups at the three collection times (T0, T5, and T20) (Table 1). Also, no differences were recorded between bees fed 0.5 and 1% enriched diets at T5 and T20 and the external controls (free-ranging nurse and guardian bees, respectively). Phenoloxidase activity significantly increased after 5 days of rearing in both the control diet and the 0.5% coconut-enriched diet group reaching levels not significantly differed from those of free-ranging nurse bees (external control). Conversely, no differences were recorded in phenoloxidase activity in bees fed a 1% coconut oil-enriched diet at T5 compared to T0. Also, no differences were recorded between T5 and T20 in the two experimental diets and when compared to the free-ranging guardian bees (external control).

In foragers, no differences were observed for any of the three investigated enzymes between the experimental and control diet groups and between T0 and T5 (Table 2).

### 3.3. Fat and Vitellogenin Contents

**Figure 4:**
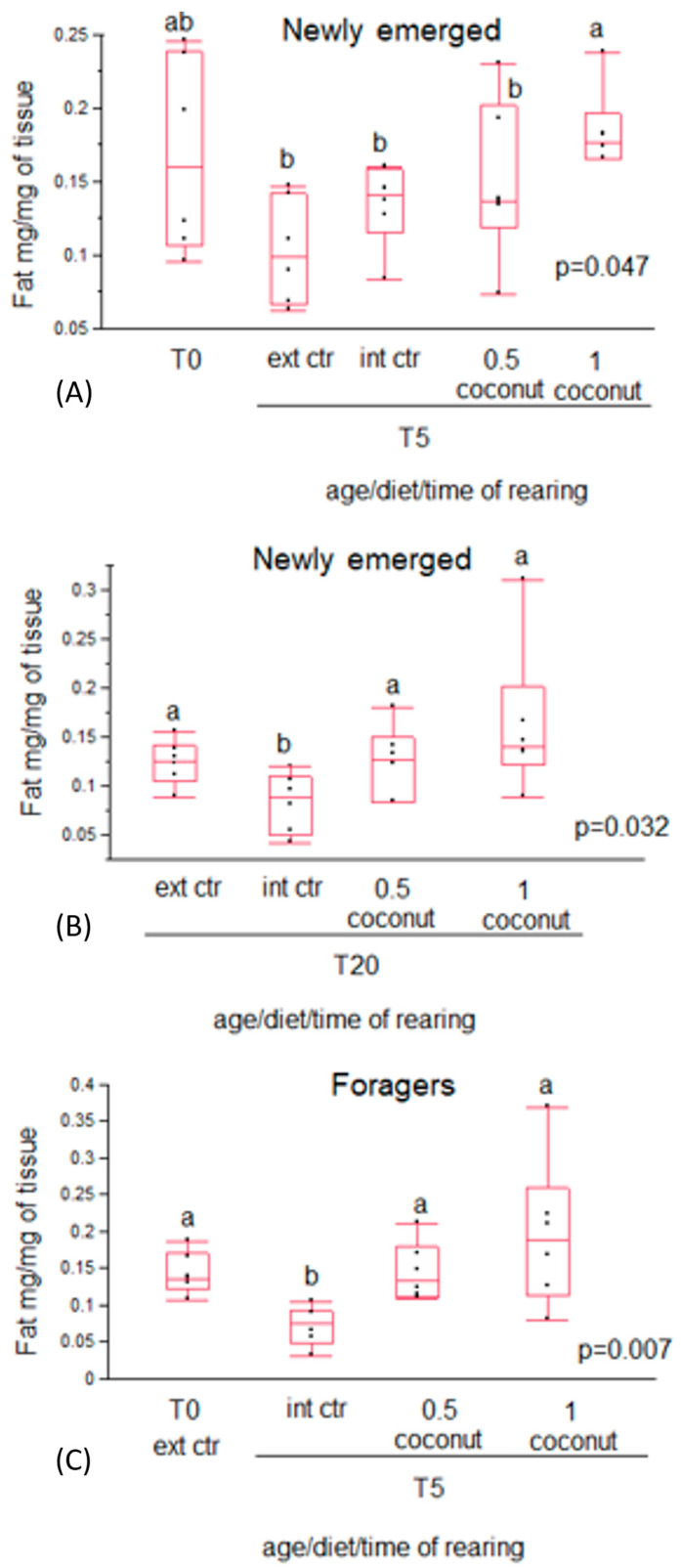
Fat content in newly emerged (**A**,**B**) and in forager (**C**) bees fed with candy (internal control diet) or with candy enriched with 0.5% and 1% coconut oil. The fat content of nurses (external control T5) and guardians (external control T20) is also reported. Different letters above the bars indicate statistically significant differences.

Newly emerged bees’ fat content at T0 was 0.168 SD 0.067 mg/mg of tissue. At T5, no differences in fat content were recorded between bees fed experimental diets, free-ranging nurse bees (external control) and T0 (Figure 4A). Bees fed a 0.5% coconut oil-enriched diet at T5 did not show differences in fat content both compared to the internal control diet group and free-ranging nurse bees (external control). Conversely, significantly higher fat content was recorded in newly emerged bees fed a 1% coconut oil-enriched diet compared to both the internal control and external control groups (Figure 4A).

On the 20th day of feeding, bees belonging to the internal control group diet showed a significantly lower fat content than at T5, and compared to those belonging to the other experimental diet groups at T20, and to free-ranging guardian bees (external control) (Figure 4B, Table 3).

In foragers bees, a significant decrease in fat content compared to T0 was recorded only in bees fed the internal control diet (Figure 4C).

In Table 4, the fat content per bee and the feed intake per bee in the different diets at the different times investigated are reported.

**Figure 5:**
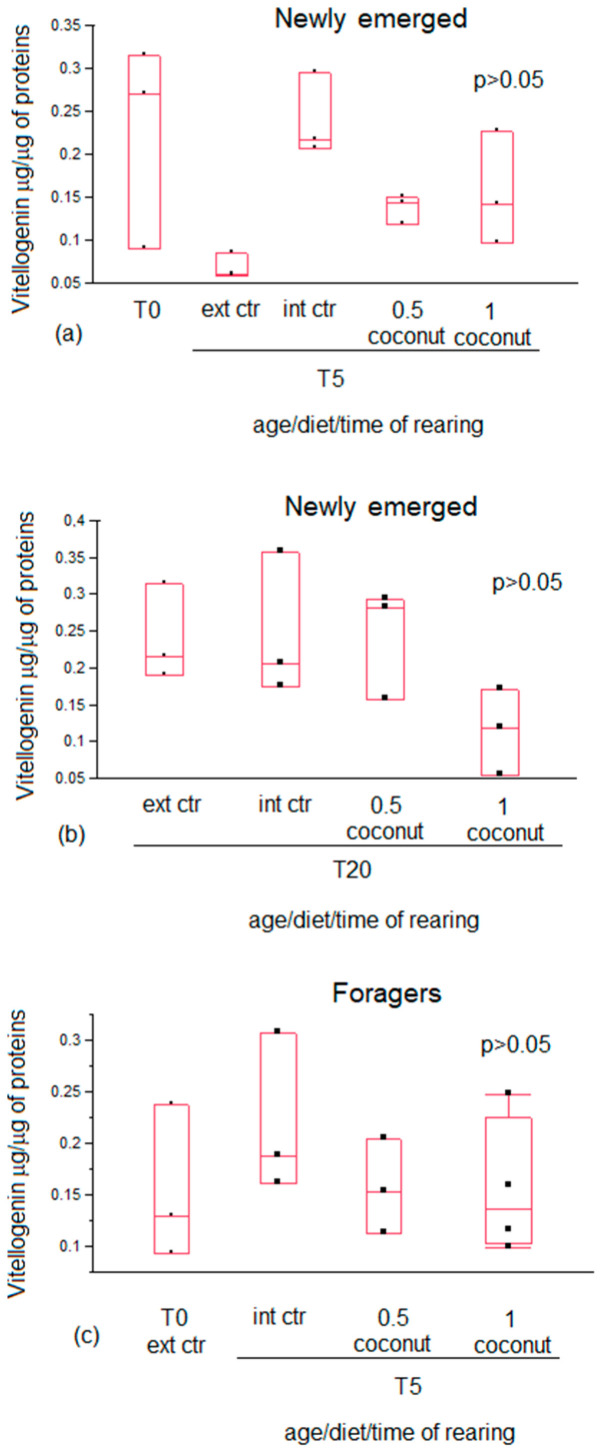
Vitellogenin content in newly emerged (**a**,**b**) and in forager (**c**) bees fed candy (internal control) or candy enriched with 0.5% and 1% coconut oil. The fat content of nurses (external control T5) and guardians (external control T20) is also reported.

In both newly emerged and forager bees, vitellogenin content did not show differences between T0 and all experimental diet groups at T5. Also, in newly emerged bees, no differences were recorded between all the experimental diet groups at T20. No differences were recorded between newly emerged diet groups and the external controls at both T5 (free-ranging nurse bees) and at T20 (free-ranging guardian bees) either. (Figure 5). A significantly lower vitellogenin content was recorded in both nurse (external control T5) bees compared to guardian (external control T20) bees and in newly emerged bees in the 0.5% coconut oil diet group at T5 compared to T20 (Table 5).

### 3.4. Correlations

Correlations among all the analysed parameters such as coconut oil intake and survival rate, glucose oxidase, phenoloxidase, glutathione S transferase, fat and vitellogenin content are reported in Table 6. In newly emerged bees, a negative correlation between phenoloxidase activity and coconut oil intake (*p* = 0.0128) on the fifth day of feeding was recorded. A positive correlation between fat content and coconut oil content was observed on both the 5th and 20th days of feeding (*p* = 0.0120 and *p* = 0.024 at T5 and T20, respectively). On the 20th day of feeding, a negative correlation was observed between vitellogenin content and coconut oil intake (*p* = 0.0185). In forager bees, no correlation was observed between coconut oil intake and the other analysed parameters.

## 4. Discussion

Supplementation with coconut oil in the bee diet resulted in a significantly lower feed intake only in newly emerged bees at an inclusion level of 1%. Conversely, a field experiment previously performed in hives by Manning et al. [31] showed that supplementation with coconut oil at 2% significantly increased pollen-enriched diet consumption without effecting bee survival compared to other tested oils. The absence of differences recorded in coconut oil consumption between bees fed diets enriched with 0.5% and 1% coconut oil suggests that for newly emerged bees, dietary fat intake is necessary to meet their physiological needs which are usually met in nature due to pollen assumption. The necessity for newly emerged bees to meet fat requirements could also explain the higher food consumption recorded in the 0.5%-enriched diet group compared to the 1%-enriched diet. A significantly higher oil consumption was recorded in foragers fed a 1% coconut oil-enriched diet than those fed a 0.5% coconut oil-enriched diet. Forager bees in nature have a sugar-based diet [33]. Therefore, since the artificial diet administered was a mixture of coconut oil and candy, the high oil consumption recorded in bees fed a 1% enriched diet could be due to higher food consumption to fill sugar requirements even though the feed intake was not significantly different. The newly emerged bees’ survival rate from the 3rd until the 15th day of feeding was positively affected by the administration of a coconut oil-enriched diet, while a reverse trend was observed from the 19th day. A reduction in the control bee group survival rate occurred in the first three days of artificial feeding; after that, it remained stable. So, as previously suggested, this result could be due to the nutritional stress caused by the lack of a fat source, which led to the survival of only those individuals that had sufficient fat reserves to mobilize for survival, confirming the key role of this nutrient in the newly emerged bee diet.

Diet enrichment with coconut oil determined differences only in phenoloxidase activity in newly emerged bees. The activity of this enzyme significantly increased from T0 to T5 only in bees fed a 0.5% coconut oil-enriched diet, remaining stable after that. Phenoloxidase activity is well known to increase according to bee age, reaching a plateau within the first week of adult life [41,42]. Therefore, the increase in phenoloxidase activity recorded could be related to this physiological trend. This hypothesis could be confirmed by the significant increase in phenoloxidase activity recorded in nurse bees (external control). In addition, the absence of an increase in phenoloxidase activity in bees fed a 1% coconut oil diet at T5 suggests an inhibitory effect of dietary coconut oil at high concentrations on the activity of this immune enzyme in young bees. A reduction in phenoloxidase activity in bees fed artificial diets (e.g., beta glucans and commercial protein diets) has been previously associated with a decrease in survival rate [16,24]. However, in the present investigation, no relationship between phenoloxidase activity and bee survival was recorded. In newly emerged bees fed a 1% coconut oil-enriched diet, a non-significant increase in phenoloxidase activity to levels comparable to those recorded in the other experimental diets was recorded on the 20th day. This result could suggest that in the long term, the inhibitory effect of a 1% coconut oil-enriched diet could be buffered by an unknown molecular mechanism. Further investigations on the daily effects of coconut oil inclusion in the bee diet on phenoloxidase activity in the first days of rearing (1–10 days) are desirable. In forager bees, no significant differences were recorded in phenoloxidase activity. The absence of differences in enzymatic activity was previously detected in foragers fed with other types (e.g., commercial protein diets) of enriched diets [24].

The administration of a coconut oil-enriched diet did not affect glucose oxidase activity in newly emerged and forager bees. This result could indicate a positive effect of coconut oil diet supplementation on bee health since an increase in glucose oxidase activity has been previously associated with a decrease in survival rate [24,25].

The absence of differences was also recorded in glutathione transferase activity in both newly emerged and forager bees. The non-alteration of glutathione transferase activity may be an indicator of a lack of feeding stress for the bee. Moreover, it could confirm that the activity of this enzyme is not induced by nutrition [24,25]. According to Chan et al. [43], a shift from lipid synthesis to lipid degradation occurs in relation to bee age. Lipid degradation determines an increase in reactive oxygen species coping by bees with a higher production of antioxidant enzymes, including glutathione transferase. Since fat content did not decrease over time in bees fed coconut oil-enriched diets, it could be hypothesized that there is an inhibition of lipid degradation due to dietary fat, resulting in a reduction in reactive oxygen species production, and, as a consequence, in antioxidant enzymes.

In newly emerged bees, the fat content at T5 was significantly higher in bees fed a 1% coconut oil-enriched diet compared to other diet groups and free-ranging nurse bees (external control). However, the fat content in free-ranging nurse bees was similar to that of bees fed the control and those with low coconut oil supplementation. Since the feed intake per bee was of the order of ten ng/bee (i.e., 0.05–0.1 ng fat ingested/bee) while the body fat content was of the order of mg/bee, it can be assumed that the extracted fat was body fat and not intestinal material. Therefore, the high fat content recorded in bees with high coconut oil supplementation could be due to an accumulation of non-degraded fat as a consequence of low motor activity in cages. A proportional increase in body fat was also observed by Stabler et al. [27] in honey bees fed artificial diets enriched with high lipid contents.

At T20, the fat content of newly emerged bees fed coconut oil at both concentrations and of free-ranging guardian bees (external control) was significantly higher than that of bees fed the internal control diet. These results allowed us to hypothesize that coconut oil determined fat gain in bees, while a sugar diet caused fat loss. In forager bees, a lower fat content in bees fed the internal control diet than in both free-ranging bees (T0 external control) and coconut oil-fed ones was recorded. This result is in accordance with Toth and Robinson [44] who observed a greater accumulation of fat in nurse bees than in foragers with a reduction in lipid levels in foragers before the onset of foraging.

In the present study, no significant differences in vitellogenin content were found among the experimental diet groups in newly emerged bees at T5 and at T20, as well as in forager bees. The vitellogenin content was significantly higher only in newly emerged bees fed 0.5% coconut oil at T20 but not at T5. A higher vitellogenin content was also recorded in guardian bees (external control for T20) compared to nurse bees (external control for T5). This result suggests that the 0.5% coconut oil diet enrichment determines a trend in vitellogenin production comparable to those trends that physiologically occur [45,46].

The fat content in newly emerged bees at both T5 and T20 was positively correlated to coconut oil intake, indicating that a higher amount of coconut oil resulted in higher fat accumulation.

However, in newly emerged bees at T5, a negative correlation between phenoloxidase activity and coconut oil intake was recorded. This negative correlation could result in a reduction in bee survival since a negative relationship between low phenoloxidase activity and bee mortality has been previously reported [16,24]. At the same time, a reduction in phenoloxidase activity subsequent to coconut oil consumption could also be a positive indicator of less nutritional stress.

In addition, since vitellogenin content should increase in foraging bees [46], the negative correlation recorded in newly emerged bees at T20 between coconut oil intake and vitellogenin content may indicate a delay in the process of aging.

## 5. Conclusions

For newly emerged bees, an artificial diet enriched with 1% coconut oil led to the following: (i) a decrease in diet consumption without a difference in coconut oil intake compared with a 0.5% coconut oil-enriched diet; (ii) an increase in survival rate from the 3rd to 14th day of feeding; (iii) a short-term (T5) decrease in phenoloxidase activity; and (iv) an increase in body fat in the long term (T20), as also recorded in the 0.5% coconut oil-enriched diet. For forager bees administered both 0.5 and 1% coconut oil-enriched diets, an increase in fat content to a level comparable to that present in free-ranging forager bees (external control) was observed. In conclusion, adding coconut oil to the diet of honey bees at low percentages (0.5% and 1%) enhances the lipid content in newly emerging and foraging bees and ultimately promotes the survival of newly emerging bees. Further investigations are needed to evaluate the use of such a supplement to prevent fat loss in weak families during winter, since the winter bees have a longer life expectancy and a slightly different physiology compared to summer bees.

## Figures and Tables

**Table 1:** Glucose oxidase, phenoloxidase and glutathione S transferase activity in newly emerged bees fed the two experimental and internal control diets (at time 0 (T0), at the 5th (T5) and at the 20th (T20) days of feeding) and in external controls (free-ranging nurse and guardian bees). Data are expressed as mean SD (median). Different letters in the same column indicate a statistically significant difference.

Newly Emerged		Glucose Oxidase Activity (U/µg of Protein)	Phenoloxidase Activity (mU/mg of Protein)	Glutathione S Transferase Activity (U/mg of Protein)
	T0	29.92 SD 25.11 (27.48)	14.78 SD 12.11 (11.07) b	24.94 SD 29.20 (15.30)
T5	External control (nurse bees)	37.31 SD 12.37 (39.27)	41.34 SD 15.85 (44.34) a	32.34 SD 8.10 (29.05)
	Internal control	32.75 SD 14.36 (31.85)	29.61 SD 11.49 (29.48) a	52.34 SD 27.73 (49.55)
	0.5% coconut	37.70 SD 18.55 (38.10)	31.11 SD 10.51 (31.28) a	33.92 SD 28.05 (26.26)
	1% coconut	30.00 SD 9.40 (30.84)	11.64 SD 8.33 (9.89) b	20.66 SD 12.98 (17.70)
T20	External control (guardian bees)	43.68 SD 9.96 (40.45)	28.06 SD 19.14 (26.49) ab	30.20 SD 20.63 (23.25)
	Internal control	27.42 SD 8.51 (29.28)	42.66 SD 23.48 (36.06) a	30.93 SD 20.98 (26.85)
	0.5% coconut	29.62 SD 11.28 (28.13)	26.93 SD 18.65 (16.96) ab	32.93 SD 20.25 (31.98)
	1% coconut	31.21 SD 9.14 (32.62)	33.63 SD 44.42 (16.77) ab	38.76 SD 22.52 (41.47)
	*p* value, χ^2^ test or F, DF = 8	*p* = 0.5922, F = 0.8163	*p* = 0.0085, χ^2^ = 20.5449	*p* = 0.4315, χ^2^ = 8.0215

**Table 2:** Glucose oxidase, phenoloxidase and glutathione S transferase activity in forager bees fed control and experimental diets (candy enriched with 0.5% and 1% coconut oil) at time 0 (T0) and at the 5th day (T5) of feeding. Data are expressed as mean SD (median).

Foragers		Glucose Oxidase Activity (U/mg of Protein)	Phenoloxidase Activity (mU/mg of Protein)	Glutathione S Transferase Activity (U/mg of Protein)
	T0 (External control)	48.33 SD 21.93 (54.94)	41.38 SD 23.77 (37.83)	38.55 SD 21.30 (29.88)
T5	Internal Control	49.37 SD 15.22 (46.71)	33.33 SD 24.37 (31.55)	35.19 SD 16.40 (35.39)
	0.5% coconut	47.93 SD 16.31 (47.76)	22.49 SD 13.66 (20.11)	19.71 SD 11.64 (21.45)
	1% coconut	30.81 SD 18.76 (26.03)	33.03 SD 32.75 (23.57)	28.96 SD 9.18 (28.89)
	*p* value, χ^2^ test, DF = 3	*p* = 0.2650, F = 1.4250	*p* = 0.5049, χ^2^ = 2.3400	*p* = 0.2360, χ^2^ = 4.2467

**Table 3:** Fat content in newly emerged bees (mg/mg of tissue): comparisons between T5 and T20 among honeybees fed candy (internal control), or candy enriched with 0.5% and 1% coconut oil and collected honeybees of the same age (external control).

Comparisons	Mean SD (Median)	*p* Value
external control T5 vs. external control T20	0.103 SD 0.036 (0.099) vs. 0.123 SD 0.023 (0.125)	*p* > 0.05
internal control T5 vs. internal control T20	0.135 SD 0.028 (0.141) vs. 0.083 SD 0.030 (0.088)	*p* = 0.0163
0.5% coconut T5 vs. 0.5% coconut T20	0.150 SD 0.054 (0.137) vs. 0.123 SD 0.037 (0.127)	*p* > 0.05
1% coconut T5 vs. 1% coconut T20	0.184 SD 0.027 (0.177) vs. 0.163 SD 0.077 (0.134)	*p* > 0.05

**Table 4:** Fat content per bee and feed intake per bee in newly emerged and forager bees at times T5 and T20, fed candy (internal control diet) or candy enriched with 0.5% and 1% coconut oil.

Time	Honeybees	Diet	Fat Content mg/bee	Feed Intake mg/bee
T5	Newly emerged	Internal control	5.050 SD 0.802	0.016 SD 0.004
T5	Newly emerged	0.5% coconut	6.267 SD 2.614	0.017 SD 0.003
T5	Newly emerged	1% coconut	5.383 SD 1.422	0.010 SD 0.002
T20	Newly emerged	Internal control	3.550 SD 1.232	0.017 SD 0.006
T20	Newly emerged	0.5% coconut	6.033 SD 2.661	0.017 SD 0.008
T20	Newly emerged	1% coconut	7.467 SD 3.189	0.010 SD 0.005
T5	Foragers	Internal control	4.650 SD 1.183	0.031 SD 0.02
T5	Foragers	0.5% coconut	5.600 SD 1.787	0.020 SD 0.011
T5	Foragers	1% coconut	7.017 SD 3.089	0.025 SD 0.011

**Table 5:** Vitellogenin content in newly emerged bees: comparisons between T5 and T20 among honeybees fed candy (internal control), or candy enriched with 0.5% and 1% coconut oil and collected honeybees of the same age (external control).

Comparisons	Mean SD (Median)	*p* Value
external control T5 vs. external control T20	0.067 SD 0.014 (0.060) vs. 0.240 SD 0.066 (0.215)	*p* = 0.0495
internal control T5 vs. internal control T20	0.239 SD 0.048 (0.217) vs. 0.245 SD 0.098 (0.206)	*p* > 0.05
0.5% coconut T5 vs. 0.5% coconut T20	0.138 SD 0.017 (0.144) vs. 0.243 SD 0.076 (0.152)	*p* = 0.0495
1% coconut T5 vs. 1% coconut T20	0.154 SD 0.066 (0.142) vs. 0.114 SD 0.058 (0.137)	*p* > 0.05

**Table 6:** Spearman correlation results among coconut oil intake and survival rate, glucose oxidase (GOX), phenoloxidase (PO) and glutathione S transferase activity, fat and vitellogenin content.

	Survival Rate	GOX Activity	PO Activity	Glutathione S Transferase Activity	Fat Content	Vitellogenin Content
Newly emerged T5 Coconut oil intake	r = 0.4426 *p* = 0.0659	r = −0.06749 *p* = 0.7902	r = −0.5737 *p* = 0.0128	r = −0.3796 *p* = 0.1202	r = 0.5779 *p* = 0.0120	r = −0.3725 *p* = 0.1279
Newly emerged T20 Coconut oil intake	r = 0.03482 *p* = 0.8909	r = 0.1668 *p* = 0.5084	r = −0.2232 *p* = 0.3733	r = 0.07580 *p* = 0.7650	r = 0.5263 *p* = 0.0249	r = −0.5483 *p* = 0.0185
Foragers T5 Coconut oil intake	r = 0.2611 *p* = 0.2953	r = −0.09746 *p* = 0.7004	r = −0.05848 *p* = 0.8177	r = −0.1906 *p* = 0.4487	r = 0.4239 *p* = 0.0796	r = −0.2239 *p* = 0.3718

## Data Availability

All data are available upon request to the corresponding author.
